# Predictive Value of Plasma Neurofilament Light Chain for Neurological Outcomes in Patients with Intracerebral Hemorrhage

**DOI:** 10.14336/AD.2023.0522

**Published:** 2024-02-01

**Authors:** Weiwei Li, Jing Wang

**Affiliations:** Department of Critical Care Medicine, Shengjing Hospital of China Medical University, Shenyang, China

**To the Editor,**

We recently read an article by Zheng et al., published in Aging and Disease [1], which suggested that plasma neurofilament light chain (NfL) is a sensitive marker for monitoring axonal injury post-intracerebral hemorrhage (ICH) and can predict both long-term functional ability and survival. Although their results are potentially valuable in clinical practice, we would like to discuss our concerns regarding their methods and results.

First, in this case-control study of 300 patients with ICH, only 41 (13.7%) patients underwent repeat NfL testing on days 7 and 14 (Table 1). The authors claimed that NfL levels were significantly higher on day 7 than at 24 h, a finding that lacks sufficient statistical strength owing to sample size limitations. Additionally, the Montreal Cognitive Assessment (MoCA) was performed in only 26 patients, accounting for less than 10% of the enrolled patients. Therefore, whether NfL levels have a predictive effect on cognitive impairment in patients after ICH remains to be determined. Furthermore, four patients had modified Rankin Scale (mRS) ≥3, so could cognitive scoring be performed?

Second, the authors concluded that the NfL level on day 7 was associated with white matter fiber integrity in post-ICH patients. However, 48.8% of these patients (Table 1) were postoperative. Do the authors need to further clarify the correlation between white matter fiber integrity and NfL levels in nonsurgical patients to exclude the effect of surgery on white matter integrity [2]? Additionally, we recommend that subsequent studies increase the sample size, and in-depth statistical analysis of data on white matter injury and cognitive function at 6 months after ICH is necessary.

Third, because the authors mentioned that plasma NfL levels on day 7 were negatively correlated with the MoCA score six months after ICH, we suggest that the authors list the specific MoCA score and classify the patient as having mild cognitive impairment or dementia according to the score, instead of simply suggesting that the plasma NfL level is related to cognitive dysfunction without mentioning the degree of impairment. Moreover, the negative effects of brain surgery on cognitive function should be elucidated [3].

Fourth, the baseline NfL levels of 300 patients with ICH were compared with those of a healthy control group. However, the age of the control group selected by the authors was significantly lower than that of the ICH group, and NfL levels are related to age [4]. Consequently, the selection of the control group was not representative. In Table 1, the age distribution of patients with NfL tested at 7 and 14 days was exactly the same as that of the 26 patients assessed using the MoCA scale. The age distributions are the same because the individuals in both groups are the same? According to the exclusion criteria of the study, patients with a history of stroke were not included. However, in Table 1, there are 35 patients with a history of cerebrovascular disease. The authors should further clarify how the exclusion criteria were applied.

Overall, the study was very interesting and well-implemented; however, we believe that the results would be more accurate and useful if the aforementioned points were addressed. Furthermore, multicenter studies should be conducted to investigate these factors on a larger scale and to validate the findings across different populations.
